# Hepatitis B Knowledge Among Iranian Adolescents: A National Survey

**DOI:** 10.5812/ircmj.11558

**Published:** 2013-12-05

**Authors:** Nader Roushan, Mohsen Nasiri Toosi, Alipasha Meysamie, Abdoul-Reza Esteghamati, Homazad Hajrassuliha

**Affiliations:** 1 Department of Internal Medicine, Division of Gastroenterology, Tehran University of Medical Sciences, Tehran, Iran; 2 Department of Preventive and Community Medicine, Tehran University of Medical Sciences, Tehran, Iran; 3 Department of Pediatrics, Tehran University of Medical Sciences, Tehran, Iran; 4 Center for Disease Control and Prevention of Iran, Tehran, Iran

**Keywords:** Hepatitis B virus, Knowledge, Health Care Surveys, Adolescent

## Abstract

**Background:**

Hepatitis B is the most prevalent chronic infectious liver disease worldwide with serious sequelae. Prevention of the infection can be provided by universal vaccination and improvement in knowledge and behavior about disease transmission. Provision of any educational program requires inquiry about target group baseline knowledge.

**Objectives:**

The aim of this study was to assess Iranian adolescents’ knowledge about hepatitis B (HBV) and associated factors.

**Patients and Methods:**

We conducted a questionnaire-based national survey of 18-year-old adolescents according to stratified cluster random sampling in Iran during 2007.

**Results:**

Response rate was 87%. Most adolescents (60%) knew that HBV infects the liver. Percentage of adolescents who gave correct answers to major routes of HBV transmission were as follows: spouse of an infected person 59%, multi-partners 66%, intravenous drug use 73%, body piercing 55% and personal belongings 55%. Higher levels of education, living in rural areas, marriage and (except for body piercing) female gender were associated with better knowledge. The knowledge of HBV infected individuals about major routes of HBV transmission was low (P < 0.001).

**Conclusions:**

There are important deficits in adolescents’ knowledge about HBV that requires attention of health educators to tailor educational programs for specific groups.

## 1. Background

Hepatitis B virus (HBV) infection may result in chronic liver disease, which can progress to important sequelae such as cirrhosis and hepatocellular carcinoma (HCC) (1). About 80% of HCC is etiologically related to HBV (2) and Asia is the home for 75% of all hepatitis B carriers (3, 4). Iran, in the Middle East, has moved from intermediate rate of HBV in the past to the current low HBV seroprevalence of 1.7% and strategies for HBV reduction is still a national public health priority (5-7). Vertical and childhood HBV transmission, once important routes of transmission in Iran (4) have decreased by widespread neonatal vaccination and screening of pregnant women in recent decades, and transmission by horizontal ways such as sexual contact and drug use, which are major ways in Western societies, have become more important (8-10).

Even though HBV infection has a lower risk of chronicity when acquired in adolescence, it is still a public health problem and 70% of HBV infections in the United States occur in adolescents (11); once infection occurs, it can be transmitted horizontally via blood products, sexual and even household contacts. Two strategies are helpful for reducing HBV infection: first, case finding and targeted education and immunization for reducing vertical, sexual and household transmission, and second, universal vaccination and promotion of safe sex practices at a community level. After graduating from high school, adolescents may go to college or start a job; in either case, they go to a more open social circumstance and would be more exposed to HBV.

It has been shown that for many diseases, having appropriate knowledge, attitude and practice may reduce the probability of disease acquisition among at risk populations. In reality, there is little knowledge about contagious diseases (12, 13) and acceptance of preventive measures could be improved by educating people (14-16). There have been several studies that evaluated the HBV knowledge of Asian minorities in the US such as Vietnamese and Cambodians in Seattle (2, 17) and Vietnamese students in Massachusetts (11) and according to the findings, the knowledge of the studied subjects was mostly low. On the other hand, studies on Singaporean people showed that their knowledge was suitable (3, 18).

## 2. Objectives

Relatively few studies have focused on Iranian adolescents. As health education programs should be based on knowledge of the target population, we conducted a community-based survey to assess Iranian adolescents’ knowledge about hepatitis B.

## 3. Patients and Methods

### 3.1. Study Design

This study was conducted during a national campaign for catch-up HBV vaccination of all 18-year-old Iranian adolescents in February 2007. The data for this study was collected using a multistage cluster sampling method from all the 30 Iranian provinces. Both rural and urban areas were included. Briefly speaking, health and medical services are provided by 41 medical sciences universities of Iran, each university in its territory has one rural health center for each village and several urban health centers for cities according to their population size. According to stratified cluster random sampling, five rural and five urban health centers were selected in each university territory; using health ministry statistics, 16,075 questionnaires were distributed among these centers proportional to their population size.

The survey was conducted by the center for disease control and prevention staff of each university in its rural and urban health centers throughout the country. The project was approved by the Research Ethics Board at the University of Tehran. To prevent any assumption of coercion, adolescents were asked to participate after being vaccinated. A brief description about the survey was given to the adolescents, after which they were invited to participate in the study. They were told that they could freely refuse to participate without any penalty. The questionnaire was self-administered and anonymous. During completion of the questionnaires, the participants were allowed to ask about the meaning of the questions, if needed. They were also convinced that their proper and correct answer to the questions is of utmost importance and may lead to highly important decisions by the health authorities that can affect their own and other people’s lives. It was also explained to them that we were interested in their knowledge, and that giving a wrong answer does not affect the study process and lead to an unfavorable result.

### 3.2. Questionnaire

All items were presented in Persian and questioned about the participants’ demographic information (place of residence, gender, marital status, education, and their father’s, mother’s and if married, spouse’s education) and knowledge. One knowledge question was about the organ infected by the hepatitis B virus, 3 questions concerned squeal of the disease and 14 questions were about the risk factors for transmission. The questionnaire was first pretested by 5 adolescents of similar age to ensure understanding of all items and then completed two times with one-week intervals, by 15 adolescents to evaluate test-retest reliability (r > 0.6). Next, it was piloted in a class of 34 students at a high school for final revision and calculation of sample size. We tried to ask about sensitive subjects with non-offensive questions (19).

### 3.3. Data Analysis

Answers to knowledge items were dichotomized into correct answers (yes or no) versus others (incorrect answer and don’t know). Proportions of respondents who correctly answered the questions were presented by percentages. Associations between demographic data and major risk factors relevant to adolescents were examined using the chi square test and logistic regression models. SPSS vs. 17.0 (SPSS Corp., Chicago, Il, USA) was used for analyses. P values < 0.05 were considered significant. Odds ratios and 95% confidence intervals were calculated for all comparisons.

## 4. Results

Response rate was 87% (13,965 questionnaires returned). More than half (57.7%) of respondents were at their appropriate level of education, which was in the last year of high school or precollege year. Table 1 shows baseline characteristics of the study population. Regarding the education levels of the respondents’ parents, only 3.1% of their mothers and 7.8% of their fathers had college degrees, and 61.9% and 49.9% of them had elementary or lower education, respectively. Nearly two-thirds of the respondents (60.3%) knew that HBV infects the liver. The lowest rate of awareness of the participants for HBV sequelae was about causation of cancer (38.1%). 

**Table 1.** Characteristics of Study Group (n = 13,964)

	Frequency of States, %
**Gender**	
Male	42.5
Female	57.5
**Residence**	
Urban	64.4
Rural	35.6
**Marital status**	
Married	8.7
Single	91.3
**Education level**	
Precollege	1.5
High school diploma	56.2
Middle school	37
Elementary school and lower	5.3
**Mother education status**	
College	3.1
High school diploma	16.5
Middle school	18.6
Elementary school and lower	61.9
**Father education status**	
College	7.8
High school diploma	22.4
Middle school	20.5
Elementary school and lower	49.4
**Spouse education status**	
College	11
High school diploma	39.5
Middle school	31.3
Elementary school and lower	18.2

The majority of respondents correctly identified blood contact and intravenous drug use (IDU) as risk factors for HBV acquisition (72.8 and 70.1 percent, respectively); the lowest rate of correct answers to risk factors was for household contact (29.2%). Table 2 shows the percentage of respondents who correctly answered the knowledge questions. Higher levels of education, living in rural areas, marriage and female gender were factors associated with better knowledge (all P values ≤ 0.03); Table 3 shows differences in knowledge about major risk factors of HBV transmission according to different variables. 

**Table 2.** Hepatitis B Knowledge

	Correct Answer	Percent
**Which organ is infected by Hepatitis B?^a^**	Liver	60.3
**Which statement is correct about Hepatitis B?**		
Hepatitis B can be severe and fatal.^b^	Yes	57
Hepatitis B can cause liver cancer.^b^	Yes	38.1
Hepatitis B can persist in body lifelong.^b^	Yes	39.7
**Who is at risk of Hepatitis B?**		
Medical personnel^b^	Yes	43.3
Prisoners^b^	Yes	59.9
Those who ingest opium.^c^	No	24.2
Those who inhale opium.^c^	No	28.4
Those who inject drugs.^b^	Yes	72.8
Those who have had contact with blood of a person with Hepatitis B.^b^	Yes	70.1
Those who have had contact with body secretions of person with Hepatitis B such as saliva^b^	Yes	47.6
Spouse of person with Hepatitis B^b^	Yes	59.1
Those who have had a person with Hepatitis B at home (except for spouse)^b^	Yes	29.2
Those who have had body piercing e.g. tattoo^b^	Yes	55.3
Those who have had hand shaking with a person with Hepatitis B^c^	No	42
Those who have breathed in a room where a person with Hepatitis B coughed or sneezed?^c^	No	40.6
Those who have shared personal belongings with a person with Hepatitis B e.g. razor (without a cut).^b^	Yes	55.2
Multi-partner persons^b^	Yes	66

^a^ Liver versus eye, skin, other organs..., Don’t know.

^b^ Yes versus No or Don’t know.

^c^ No versus Yes or Don’t know.

**Table 3.** Relationship Between Independent Variables and Knowledge About Major Risk Factors of HBV Transmission Relevant to Adolescents^a ^

Demographics	Major Risk Factors for HBV Transmission				
	Sexual Contact		Intravenous Drug Use	Body Piercing	Personal Belongings
	Spouse of an Infected Person	Multi-Partnership			
**Gender**					
Male	55.7 ^**^	63.4 ^**^	71.4 ^**^	54.5	54.8 ^**^
Female	61.5	68.1	73.9	55.9	55.5
OR (95%CI)^b^	1.24 (1.1-1.4)	1.26 (1.1-1.5)	1.37 (1.2-1.6)	0.96 (0.9-1.1)	0.78 (0.7-0.9)
**Residence**					
Urban	58.6 ^*^	65.6 ^**^	70.8 ^**^	52.4 ^*^*	52.3 ^**^
Rural	60.1	67.1	76.8	60.7	60.2
OR (95% CI)	1.14 (1.0-1.3)	1.5 (1.3-1.8)	1.4 (1.3-1.6)	1.4 (1.2-1.6)	1.25 (1.1-1.4)
**Marital status**					
Married	61.3 ^**^	68.3 ^**^	75.9 ^**^	58.2 ^**^	57.7 ^**^
Single	59	66	72.5	55.1	55
OR (95% CI)	2.1 (1.7-2.5)	1.2 (1.0-1.4)	1.16 (1.0-1.3)	1.5 (1.2-1.8)	1.2 (1.1-1.4)
**Education**					
Precollege	76.9 ^**^	80.9 ^**^	87 ^**^	66.5 ^*^	61.1 ^*^
High school diploma	61.1	67.9	73.3	55.6	54.7
Middle school	56.9	64.4	72.4	55.1	56.9
Elementary school and lower	50	57.6	68.1	53.1	52.5
OR (95% CI)	0.88 (0.8-0.9)	0.82 (0.7-0.9)	0.88 (0.8-0.9)	0.96 (-)	0.99 (-)
**Mother education**					
College	64.3 ^**^	69.1 ^*^	72.2 ^*^	65.1 ^**^	53.9
High school diploma	62.9	69.1	74.5	59	56
Middle school	60.4	67.5	72.7	53.7	54.8
Elementary school and lower	57.6	64.9	72.4	54	55.4
OR (95% CI)	1.1 (1.0-1.2)	1.1 (1.0-1.2)	1.1 (1.0-1.1)	1.14 (1.1-1.2)	1.0 (0.97-1.1)
**Father education**					
College	65.8 ^**^	69.7 ^*^	75.3	63.2 ^**^	55.9
High school diploma	61.9	67.8	73.2	55.8	54.8
Middle school	59.5	65.6	72	52.5	54.1
Elementary school and lower	56.6	65.1	72.4	54.6	55.8
OR (95% CI)	1.2 (1.1-1.2)	1.1 (1.0-1.2)	1.1 (1.0-1.2)	1.1 (1.1-1.2)	1.0 (0.99-1.1)
**Spouse education**					
College	75.6 **	81.9 **	83.6	68.3	55.1
High school diploma	63.6	72.7	76.3	57.7	58.7
Middle school	58.6	66.3	74.1	55.7	57.1
Elementary school and lower	52.7	56.9	72.1	58.1	56.9
OR (95% CI)	1.4 (1.2-1.7)	1.6 (1.3-2.1)	1.1 (0.8-1.5)	1.2 (0.9-1.5)	0.99 (0.84-1.2)

^a^ Numbers refer to the percentages of the respondents who correctly answered about risk factors of HBV transmission.

^b^ odds ratio (95% confidence interval).

^*^ Asterisks refer to those associations that are significant (P value < 0.05 * and < 0.001 **).

## 5. Discussion

We found that knowledge of Iranian adolescents were different from the rest of the Asian population. Since no previous study used the same questionnaire as ours, we inevitably compared our study with several of them. The number of Iranians who knew that HBV infects the liver was more than Vietnamese students (11) (60.3 vs. 23 percent) while Iranians also had greater knowledge about the statement: “infection could persist in the body lifelong” when compared to Cambodian women (2) (39.7 vs. 24 percent). But regarding carcinogenicity of HBV, their knowledge was lower than Cambodian women (2) and Singaporean people (3) (38.1 vs. 54 and 86 percent respectively).

Sexual contacts, IDU, body piercing and personal belongings are four important risk factors relevant to adolescents. We addressed sexual contact as a risk factor by asking whether spouse of an infected person or a person with multi-partners was at risk (correct answer to these questions: 59.1 and 66 percent, respectively); although their knowledge was not so different from responses from Cambodian ( 2 ) and Vietnamese people ( 17 ) (47 and 69.5 percent, respectively), it was still low and as this is one of the important routes of HBV transmission, it requires special consideration in health education programs. As shown in Table 3, females, married people, residents of rural areas and higher educated respondents or those with higher educated mother or father were more aware of sexual contact as a risk factor. 

Knowledge about the risk of IDU, an emerging problem in recent decades in Iran (20), was acceptable and comparable to that of Singaporean people (72.8 vs. 74 percent) (3). Body piercing similar to tattooing, popular nowadays, (21) carries risk of HBV infection if the needles are shared without disinfection. In a study of undergraduate students at the health campus of a US university (22), 20% addressed hepatitis B as a risk of body piercing which is lower than the response rate among Iranians (55.3%). One of the risk factors of HBV transmission that often goes unnoticed is the use of common personal belongings such as razors and nail cutters in hairdressings and manicures. Only 55.2% of adolescents were aware of this risk factor and the response rate was lower compared to that of Vietnamese people (17) and that of Chinese people in Canada (63, 68 percent for razor, respectively) (23).

The relationship between level of education and knowledge about HBV has been shown by previous studies ( 3 , 4 ). Adolescents with higher education are more likely to have heard or read about HBV in schools or through media. Another implication of this relationship may be that, further health education should be aimed at those with lower education. We found that males usually had less knowledge about major risks of HBV transmission and since a previous health survey in Iran showed ( 4 , 24 ) that seroprevalence of HBV was higher in males (1.9 vs. 1.5 percent in female), this group should be the focus of greater education. In our study 7.3% of respondents had been tested for HBV; they probably had felt at risk of HBV, so they had gone for the test. Unfortunately, this group and HBV positive people had lower levels of knowledge about major risks of HBV transmission and this can promote further HBV propagation (Table 3). This was in contrast to findings of a study about knowledge of HBV infected people in Singapore which revealed that they had a good knowledge of HBV ( 18 ). 

Regarding the ways which do not result in transmission of HBV, Iranian adolescents’ knowledge about hand shaking was lower than Cambodian (2) and Vietnamese (17) people (42 vs. 69 and 75 percent, respectively) but higher than Cambodian and Vietnamese about Coughing (40.6 vs. 10 and 31 percent, respectively). Although this knowledge doesn’t seem to be important, if misperception of community is not addressed, it can lead to stigmatization of HBV infected patients and may result in them hiding their disease in situations that requires clear stating of their HBV positivity such as dentistry. Our study had several limitations. Due to restrictions with questioning people’s sexual activity, we couldn’t inquire the pattern of sexual activity of adolescents such as the use of condoms. All data were self-reported and we couldn’t validate our data about testing for HBV or HBV positivity. On the other hand, it is possible that adolescents have had difficulty in understanding the exact meaning of questions. Nevertheless, results of this study can be used for hepatitis B preventive programs by public health and medical professionals. Future studies can focus on young people’s behavior, especially high-risk groups such as HBV positive people and IV drug users and also the situation of hair dressing salons.

In conclusion, our study suggests that there were several deficits in adolescents’ knowledge about HBV. These deficits were more prominent among less educated people, urban inhabitant, single people and males. HBV infected people, a source of HBV propagation, had low knowledge about the routes of transmission.
